# Encoding tasks moderated the reward effect on brain activity during memory retrieval

**DOI:** 10.1038/s41598-022-12344-9

**Published:** 2022-05-17

**Authors:** Qianqian Ding, Jinfu Zhu, Chunping Yan

**Affiliations:** College of Psychology, Xinxiang Medical University, Xinxiang, China

**Keywords:** Neuroscience, Psychology

## Abstract

Previous studies have explored the effects of retrieval reward and depth of processing in encoding on recognition, but it remains unclear whether and how reward and depth of processing during encoding influence recognition. We investigated the effect and neural mechanisms of encoding reward and processing depth on recognition using event-related potentials (ERPs) in this study. In the study phase, participants were asked to perform two encoding tasks: congruity-judgment (deep processing) and size-judgment (shallow processing) in reward and no-reward conditions. The test phases included object (item) and background (source) tests. The results of item retrieval showed that the accuracy of rewarded items was higher than that of unrewarded items only in the congruity-judgment task, and the reward effect (the average amplitudes in the reward condition were significantly more positive than those in the no-reward condition) in the 300–500 and 500–700 ms were greater in the congruity-judgment task than in the size-judgment task. The results of source retrieval showed that the accuracy of rewarded items was higher than that of unrewarded items, that the difference in the size-judgment task was significantly larger, and that the reward effect in the 300–500 and 500–700 ms were greater in the size-judgment task than in the congruity-judgment task. In conclusion, the encoding task moderated the reward effect in item and source memory.

## Introduction

Previous studies have shown that incentive motivation occurs when our behavior is directed toward a valued goal^1–6^. Monetary rewards can adjust top-down attention, bias attention to and promote the processing of reward-related stimuli. Reward anticipation improves episodic memory performance. Episodic memories are characterized by both content and context. If a task requires a participant to merely determine whether the stimulus was present at a particular time, that task is assessing item memory. If a task requires participants to recall the information associated with the stimulus, that task is assessing source memory^7–9^. Episodic memory is also affected by other factors. For example, the levels-of-processing theory suggests that the task nature in the encoding phase has different effects on memory retrieval and that deeper processing strategies enhance memory performance^1,5^. Recent works have also found that people learn to selectively engage deep encoding strategies to enhance memory of high-value information^10–12^. In addition, a previous study found that the reward effect in item memory retrieval was also affected by encoding tasks^13^.

Shigemune et al. investigated the effects of reward and encoding strategies on item memory using fMRI techniques, and they asked participants to encode words using either deep or shallow strategies, which led to variation in the difficulty of subsequent retrieval; during retrieval, participants selected target words under either high or low monetary reward conditions^13^. A reward-related enhancement of memory was found only when memory retrieval was difficult (corresponding to shallow encoding)^13^, which was consistent with previous findings of stronger reward effects during more difficult memory retrieval tasks^14–16^. fMRI scans have shown that reward-related increases in activation of the substantia nigra/ventral tegmental area (SN/VTA), medial temporal lobe (MTL), dorsomedial prefrontal cortex (dmPFC), and dorsolateral prefrontal cortex (dlPFC) were greater during the difficult than those that were easier to retrieve, and their result can be explained by the optimal level theory, which states that subjective motivations and preferences are highest when stimulus variables such as complexity, novelty, uncertainty, conflict, and difficulty are maintained at an optimal level^13,17–20^. For example, the motivation role to complete tasks at an appropriate level of difficulty is stronger than that of tasks that are too easy or difficult to encode, suggesting that if we want to complete specific tasks and requirements, we should ensure that the tasks are sufficiently complex and novel. Unlike the study by Shigemune et al., they only explored item memory, and source memory was also explored in our study because it is used more often in our daily life; therefore, we investigated the effects and neural mechanisms of encoding reward and processing depth on item memory and source memory, utilizing event-related potential (ERP) measurements. In our study, ERPs were used because they have high temporal resolution and can better assess a temporal stream of neural activity than fMRI measurements, and we wanted to further verify the temporal characteristics of the effects as well as the neural mechanisms of reward and processing depth during encoding on item and source memory retrieval.

ERP studies have shown that LPP (a late positive component that appears approximately 300 ms after stimulus onset) reflect the effect of reward motivation and emotion^3,21^. ERP studies on memory have also shown significant reward effects in the late LPP (the average amplitudes in the reward condition were significantly more positive than those in the no-reward condition) in test^2–4^. The duration of later LPP might reflect stimulus significance and memory encoding^22–24^. Previous ERP studies have shown that the average amplitudes of items with deeper processing strategies were more positive than those of items with shallower processing strategies^25^. In addition, previous ERP studies have shown that the average amplitudes of old items were more positive than those of new items for both components, a finding called the “old/new effect”^26–28^. Dual-process theory proposes that familiarity (when individuals sense that the item is familiar but cannot recall any details about it) and recollection (when people recall related details about an item) are dissociable processes contributing to recognition memory^8,27^. The FN400 old/new effect (an earlier frontal negative component that peaks at approximately 400 ms after stimulus onset) correlates with familiarity; the LPC old/new effect (a late parietal positive component that peaks at approximately 600 ms after stimulus onset) correlates with recollection^26–28^.

Following a previous study^11^, two encoding tasks (a congruity-judgment task and a size-judgment task) to vary the depth of processing were used during encoding. The congruity-judgment task involved determining whether the object image matched the background image (object either always occurred in the background or often was used in the background in real life. For example, in a basketball and basketball court, basketball is always on basketball court), and the size-judgment task was to judge the size of the actual object compared to the size of the computer screen. As determined by previous studies, the congruity-judgment task involves deep processing, and the size-judgment task involves shallow processing^29,30^. In our current study, during encoding, participants were required to remember images (half of the images were rewarded) while carrying out the two encoding tasks. Object and background tests were separately conducted immediately after the study phase, which ensured that old items judged to be “new” also could be judged as the source^31^. During the object test phase, participants were required to judge whether the object image was new or old, which indicated item memory. Then, participants were asked to select the background that matched the object during the background test phase, which indicated source memory.

Based on previous studies, we predicted that for item memory, the accuracy of rewarded items would be significantly higher than that of unrewarded items in the congruity-judgment task and that the significant reward effects of FN400 and LPC in the congruity-judgment task would be greater than those in the size-judgment task. For source memory, we predicted that the accuracy of rewarded items would be significantly higher than that of unrewarded items in the size-judgment task and that the significant reward effect of FN400 and LPC in the size-judgment task would be greater than that in the congruity-judgment task as well as appearing earlier than in item memory.

## Materials and methods

### Participants

We recruited thirty right-handed college students (mean age = 21.5 years; 14 of whom were male) from Xinxiang Medical University. The sample size was determined based on results of a power analysis run using G-Power sofware^32^ (power > 0.8, *α* = 0.05), which indicated that a minimum sample size of *n* ≥ 18 was required for that purpose. All participants had normal or corrected-to-normal vision and no history of neurological or psychiatric disorders. Three participants were excluded from the background test ERP analysis because they did not reach our ERP analysis criterion of at least 16 trials per condition (the three participants’ number of trials during the no-reward condition in the size-judgment task: 14 trials; 14 trials; 13 trials), resulting in a final sample of 27 participants (mean age = 20.9 years; 13 of whom were male). All participants provided written informed consent for their participation. This study was approved by the Xinxiang Medical University Human Research Committee, and all methods were performed in accordance with its relevant guidelines and regulations. The participants received monetary compensation after the experiment.

### Materials

The target stimuli consisted of 640 color neutral images (320 background images, 320 object images) and were selected from the Chinese Affective Picture System (CAPS)^33^, the International Affective Picture System (IAPS)^34^ and the internet. All background images (688 × 510 pixels) and object images (433 × 310 pixels) were uniform in size. Twenty-two college students (12 male) who did not attend the formal experiment provided valence (1 = very not happy, 9 = very cheerful) and arousal (1 = very calm, 9 = very excited) ratings of these images. The average valence and arousal scores of the object images were 5.03 ± 0.46 and 4.07 ± 0.55, respectively, and those of the background images were 5.20 ± 0.47 and 4.33 ± 0.55, respectively. Of these images, 160 object images and 160 background images were used as study (old) items, and another 160 object images and 160 background images were used as test (new) items. Study and test items were matched on valence ratings [object: old (5.03 ± 0.48) and new (5.02 ± 0.44); background: old (5.24 ± 0.44) and new (5.17 ± 0.50)] and arousal ratings [object: old (4.11 ± 0.54) and new (4.03 ± 0.55); background: old (4.32 ± 0.54) and new (4.34 ± 0.56)]. The object and background images were divided into four groups, each group containing 40 images; the four groups of object or background images were matched on valence and arousal (see Table 1).

**Table 1:** Average valence and arousal ratings of image groups in the study phase.

	Images type	Group 1 (*n* = 40)	Group 2 (*n* = 40)	Group 3 (*n* = 40)	Group 4 (*n* = 40)	*F*_(3, 156)_	*p*
Valence	Object	5.03 ± 0.08	5.13 ± 0.07	5.03 ± 0.06	4.94 ± 0.08	1.04	0.376
	Background	5.29 ± 0.08	5.13 ± 0.07	5.26 ± 0.07	5.29 ± 0.06	1.26	0.291
Arousal	Object	4.23 ± 0.10	4.12 ± 0.08	3.99 ± 0.07	4.06 ± 0.09	1.41	0.242
	Background	4.35 ± 0.08	4.27 ± 0.10	4.22 ± 0.09	4.45 ± 0.06	1.46	0.228

The data after “ ± ” in the table are the standard errors of the mean.

In addition, 20 neutral object and 20 neutral background images were selected from IAPS as training materials; the training images did not appear in the formal experiment.

### Procedures

The experimental program was compiled via Presentation. Before the formal experiment, the participants were familiarized with the experimental procedure and keystroke responses through practice. The participants were told that in the object test phase, they would obtain a monetary reward (RMB 0.20) for each correctly recognized and judged old object in the study phase; however, they would lose RMB 0.10 for each new object incorrectly marked as old. Unrewarded objects were not rewarded or punished for being correctly or incorrectly judged. In the background test phase, participants received RMB 0.20 if the original background of an old object was correctly selected in the reward condition; the cumulative cash payment was allocated at the end of the experiment.

**Figure 1:**
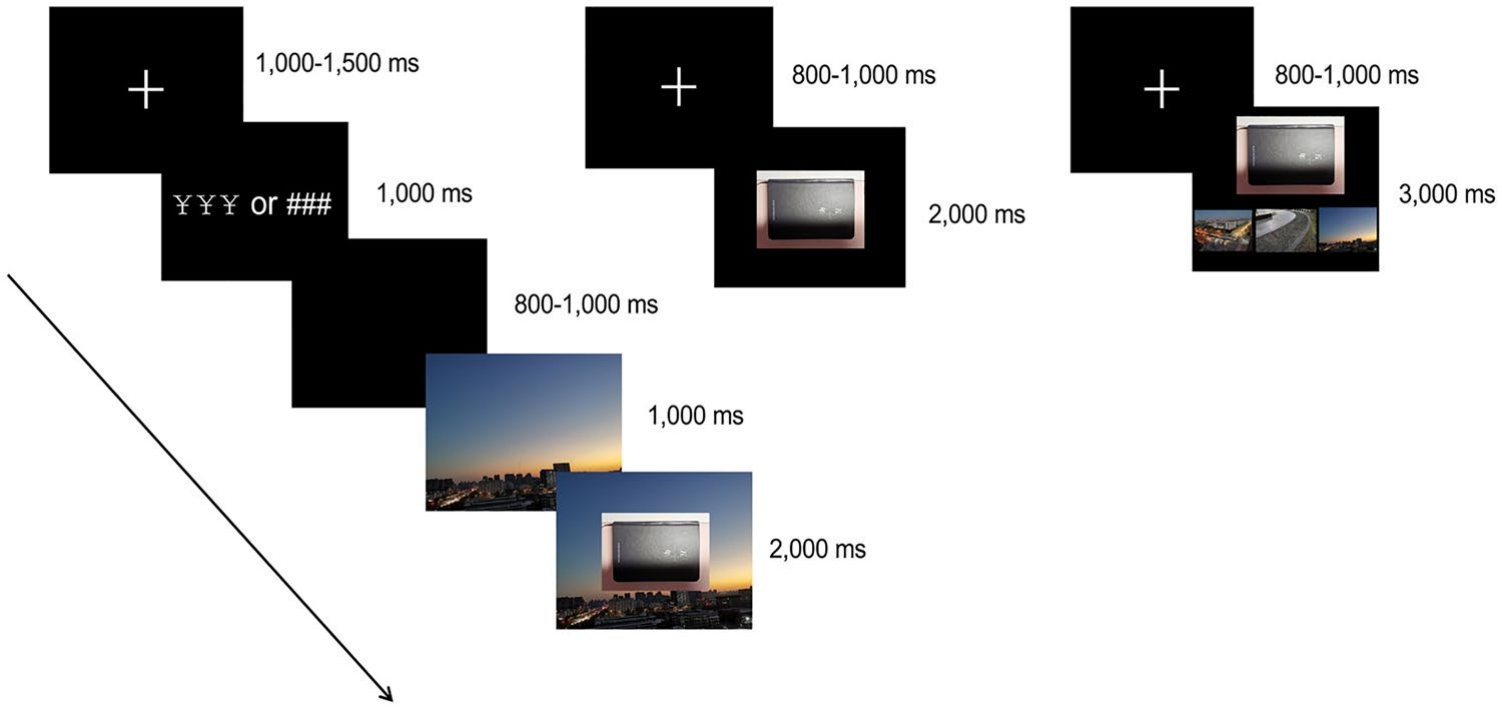
Schematic representations of a trial in the study phase (left), object test phase (middle) and background test phase (right). See text for details. Due to copyright restrictions all original images have been replaced with images taken by the authors.

The formal experiment included 4 blocks (2 congruity-judgment tasks, 2 size-judgment tasks), each containing a study phase, an object test phase and a background test phase (see Fig. 1). In the study phase, each trial began with a cross fixation point for 1000–1500 ms, followed by a reward cue (¥ ¥ ¥) or nonreward cue (# # #) for 1000 ms, and then a blank screen was presented for 800–1000 ms. After that, a background image was presented for 1000 ms. Then, an object image was superimposed in the middle of the background image presented together for 2000 ms, during which the participants were asked to perform the corresponding task (congruity-judgment or size-judgment task). After the study, participants were asked to perform a distraction task concerning subtraction from an initial three-digit number (i.e., repeatedly subtracting 3 from each number) for 1 min. After that, the test phases (object and background) were carried out. In the object test phase, each trial began with a cross fixation point presented for 800–1000 ms, and then the object image was presented for 2000 ms. Participants judged whether the object image was new or old. Next, in the background test phase, each trial began with a cross fixation point presented for 800–1000 ms, and then 4 images [the top image was an old object image, and the bottom images were three background images (original, reconstructed and new)] were presented for 3000 ms during which participants selected the background that matched the object from the three background images. The order of the trials was pseudorandom and sequential. All of the reaction keys were counterbalanced between the left and right hands across the participants. The order of the four blocks was also counterbalanced across participants.

### ERP recordings and analysis

Electroencephalographic (EEG) data were recorded by a 64-channel Neuroscan system at a 500 Hz sampling rate with a 0.1–100 Hz bandpass filter. The reference electrode was placed on the left mastoid process, and the connection point was midway between FPz and Fz. These electrode locations conform to the extended international 10–20 system. Electrooculogram (EOG) was recorded with two pairs of electrodes, one pair placed above and below the left eye and another pair at the outer canthi of both eyes. All electrodes were referenced online to the left mastoid and rereferenced offline to the average of the right and left mastoid recordings. EOG blink artifacts were corrected using a linear regression estimate^35–37^. EEG/EOG signals (impedance < 5 kΩ) were digital bandpass filtered at 0.05–40 Hz and corrected to a 200 ms prestimulus baseline. Trials with a voltage exceeding ± 100 µV were excluded from the ERP analysis.

In this study, ERP data were used to analyze EEG changes in the three phases: the study, object test and background test phases. The time ranges of EEG analysis were 2000 ms (study phase) and 1000 ms (test phases). ERPs were analyzed from five representative midline electrodes (Fz, FCz, Cz, CPz, and Pz), where the effects of condition were most evident and aligned with the findings of previous studies^38^ that used similar experimental designs and reported such effects. Intervals were selected for the study phase (500–700 ms, 700–1200 ms and 1200–1700 ms), and the object test and the background test phases (300–500 ms and 500–700 ms). These intervals were selected based on visual inspection of grand-average ERPs, given that similar intervals have been used in prior studies^3,26,39^ of related ERP phenomena (FN400, LPC and LPP).

The effective trials under the four conditions in the encoding phase: 37–40 trails, 33–40 trails, 35–40 trails, and 31–40 trails; under the six conditions in the item retrieval phase: 21–40 trails, 19–40 trails, 54–79 trails, 19–37 trails, 20–38 trails, and 60–79 trails; under the four conditions in the source retrieval phase: 25–38 trails, 24–36 trails, 18–33 trails, and 17–28 trails. Repeated-measures ANOVAs were corrected using the Greenhouse–Geisser method. The alpha level was 0.05. Multiple comparisons and simple effect analyses were corrected using Bonferroni correction. All data analyses were conducted with SPSS software.

## Results

### Behavioral data

**Figure 2:**
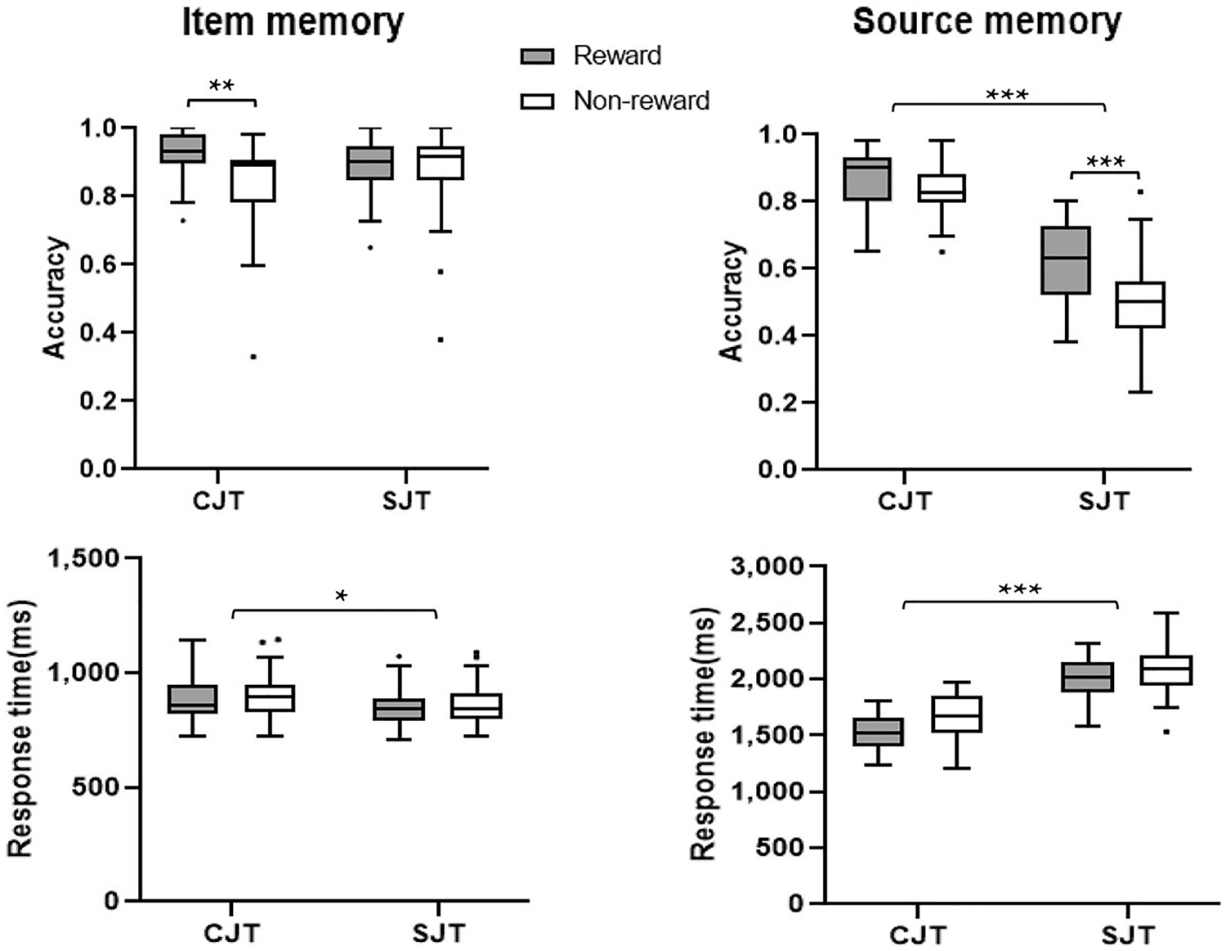
Behavioral performance across conditions for item memory and source memory. CJT, congruity-judgment task; SJT, size-judgment task. *p < 0.05, **p < 0.01, ***p < 0.001.

A two-way, 2 (encoding task: congruity-judgment task vs. size-judgment task) × 2 (reward type: reward vs. no-reward) repeated-measures ANOVA was conducted on the accuracy (ACC) and response time (RT) of the test phases. Participants’ recognition performances in both item and source memory are given in Table 2 and Fig. 2.

**Table 2:** Average ACC and RT of the test phases.

Test type	Dependent variable	Congruity-judgment task		Size-judgment task	
		Reward	Non-reward	Reward	Non-reward
Object test	ACC	0.92 ± 0.01	0.84 ± 0.02	0.88 ± 0.02	0.87 ± 0.02
	RT (ms)	880.09 ± 18.43	902.13 ± 19.15	851.21 ± 15.84	864.80 ± 16.17
Background test	ACC	0.86 ± 0.02	0.82 ± 0.01	0.62 ± 0.02	0.51 ± 0.03
	RT (ms)	1,528.39 ± 31.99	1,672.76 ± 36.37	2,006.91 ± 31.98	2,088.45 ± 40.81

The data after “ ± ” in the table are the standard errors of the mean.

### Behavioral data in the object (item) test phase

ANOVA of the ACC revealed a significant main effect of reward type [*F*_(1, 29)_ = 5.79, *p* = 0.023, *η*_*p*_^2^ = 0.17], no significant main effect of encoding task [*F*_(1, 29)_ = 0.00, *p* = 1.000, *η*_*p*_^2^ = 0.00], and a significant reward type × encoding task interaction [*F*_(1, 29)_ = 10.24, *p* = 0.003, *η*_*p*_^2^ = 0.26]. Further simple effect analysis found that the accuracy of rewarded items (*M* = 0.92, *SD* = 0.06) was significantly higher than unrewarded items (*M* = 0.8, *SD* = 0.13) (*p* = 0.001) in the congruity-judgment task, but there was no significant difference between rewarded (*M* = 0.88, *SD* = 0.08) and unrewarded items (*M* = 0.87, *SD* = 0.13) in the size-judgment task (*p* = 0.554).

ANOVA of the RT revealed significant main effects of reward type and encoding task [*F*_(1, 29)_ = 6.17, *p* = 0.019, *η*_*p*_^2^ = 0.18; *F*_(1, 29)_ = 7.26, *p* = 0.012, *η*_*p*_^2^ = 0.21] but no reward type × encoding task interaction [*F*_(1, 29)_ = 0.40, *p* = 0.535, *η*_*p*_^2^ = 0.01]. The response times of rewarded items (*M* = 865.65, *SD* = 94.46) were faster than thode of unrewarded items (*M* = 883.46, *SD* = 98.07) (*ps* < 0.05), and the response time of the size-judgment task (*M* = 858.00, *SD* = 87.19) was faster than that of the congruity-judgment task (*M* = 891.11, *SD* = 102.68) (*ps* < 0.05).

### Behavioral data in the background (source) test phase

ANOVA of the ACC revealed a significant main effect of reward type and encoding task [*F*_(1, 29)_ = 35.84, *p* < 0.001, *η*_*p*_^2^ = 0.55; *F*_(1, 29)_ = 204.30, *p* < 0.001, *η*_*p*_^2^ = 0.88] and a significant reward type × encoding task interaction [*F*_(1, 29)_ = 5.43, *p* = 0.027, *η*_*p*_^2^ = 0.16]. Further simple effect analysis showed that the accuracy of the congruity-judgment task (*M* = 0.84, *SD* = 0.08) was significantly higher than that of the size-judgment task (*M* = 0.56, *SD* = 0.14) in both reward and no-reward conditions (*ps* < 0.001); the accuracy of rewarded items (*M* = 0.74, *SD* = 0.16) was significantly higher than unrewarded items (*M* = 0.67, *SD* = 0.19) in both the congruity-judgment and size-judgment tasks (*ps* < 0.001). The reward difference (reward minus no reward) between the two encoding tasks was further calculated by a paired-samples *t* test, which showed that the reward difference of the size-judgment task was significantly greater than that of the congruity-judgment task [*t*_(29)_ = − 2.35, *p* = 0.026].

ANOVA of the RT revealed significant main effects of reward type and encoding task [*F*_(1, 29)_ = 33.55, *p* < 0.001, *η*_*p*_^2^ = 0.54; *F*_(1, 29)_ = 179.72, *p* < 0.001, *η*_*p*_^2^ = 0.86], but no reward type × encoding task interaction [*F*_(1, 29)_ = 3.54, *p* = 0.070, *η*_*p*_^2^ = 0.11]. Further multiple comparisons analysis indicated that the response time of rewarded items (*M* = 1767.65, *SD* = 297.29) was faster than unrewarded items (*M* = 1880.60, *SD* = 296.65) (*ps* < 0.001) and that the response time of the congruity-judgment task (*M* = 1600.58, *SD* = 199.73) was faster than the size-judgment task (*M* = 2047.68, *SD* = 203.30) (*ps* < 0.001).

### ERP data

#### ERPs in the study phase

**Figure 3:**
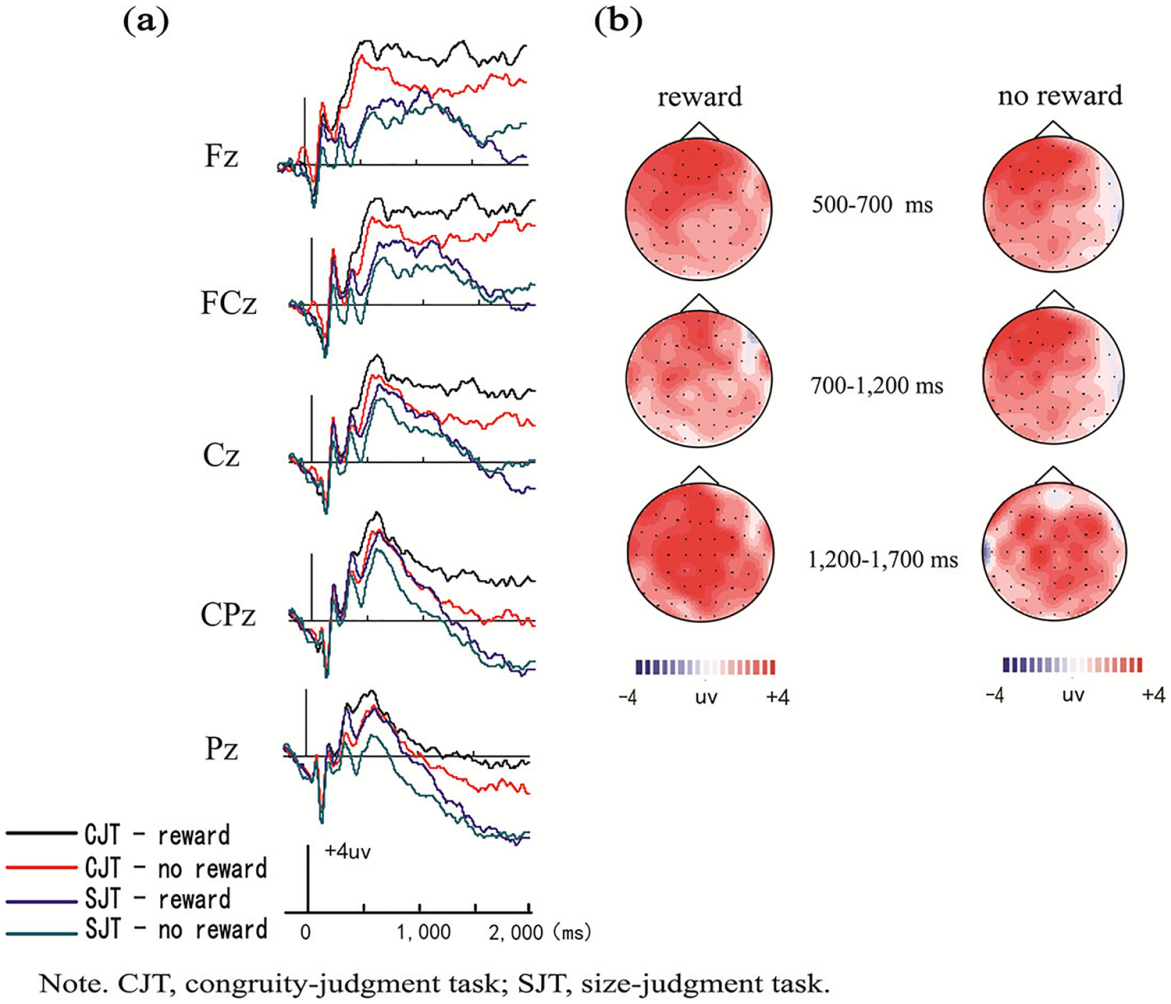
Amplitude distribution and topographic maps of ERPs in the different conditions in the study phase. (**a**) Comparison of ERP amplitudes in the four conditions. (**b**) Topographic maps of encoding effects ERPs (congruity-judgment task minus size-judgment task) both in reward and no-reward conditions.

Average amplitudes for each condition during each time window were analyzed by 2 (encoding task: congruity-judgment task vs. size-judgment task) × 2 (reward type: reward vs. no-reward) × 5 (electrode location: Fz, FCz, Cz, CPz, and Pz) repeated-measures analyses of variance (ANOVAs). The amplitude distribution and topographic maps of ERPs in each phase are given in Fig. 3.

##### LPP (500–700 ms)

ANOVA revealed significant main effects of reward type, encoding task and electrode location [*F*_(1, 29)_ = 13.69, *p* = 0.001, *η*_*p*_^2^ = 0.32; *F*_(1, 29)_ = 13.55, *p* = 0.001, *η*_*p*_^2^ = 0.32; *F*_(4, 26)_ = 26.55, *p* < 0.001, *η*_*p*_^2^ = 0.80] and a significant electrode location × encoding task interaction [*F*_(4, 26)_ = 8.28, *p* < 0.001, *η*_*p*_^2^ = 0.56]. Further simple effect analysis found that the average amplitudes of the congruity-judgment task were more positive than those of the size-judgment task, but only at electrodes Fz, FCz and Pz (*ps* < 0.01).

##### LPP (700–1200 ms)

ANOVA revealed significant main effects of reward type, encoding task and electrode location [*F*_(1, 29)_ = 15.25, *p* = 0.001, *η*_*p*_^2^ = 0.35; *F*_(1, 29)_ = 14.91, *p* = 0.001, *η*_*p*_^2^ = 0.34; *F*_(4, 26)_ = 18.49, *p* < 0.001, *η*_*p*_^2^ = 0.74] and a significant three-way interaction among encoding task, reward type and electrode location [*F*_(4, 26)_ = 4.39, *p* = 0.008, *η*_*p*_^2^ = 0.40]. Further simple effect analysis showed that the average amplitudes of the congruity-judgment task were more positive than those of the size-judgment task at electrodes FCz and Pz in reward conditions (*ps* < 0.05).

##### LPP (1200–1700 ms)

ANOVA revealed significant main effects of reward type, encoding task and electrode location [*F*_(1, 29)_ = 16.51, *p* < 0.001, *η*_*p*_^2^ = 0.36; *F*_(1, 29)_ = 143.19, *p* < 0.001, *η*_*p*_^2^ = 0.83; *F*_(4, 26)_ = 35.61, *p* < 0.001, *η*_*p*_^2^ = 0.85] and a significant reward type × encoding task interaction [*F*_(4, 26)_ = 10.36, *p* = 0.003, *η*_*p*_^2^ = 0.26]. Further simple effect analysis found that the average amplitudes of the congruity-judgment task were more positive than those of the size-judgment task at all electrodes, but only in reward conditions (*p* < 0.001).

#### ERPs in the object (item) test phase

**Figure 4:**
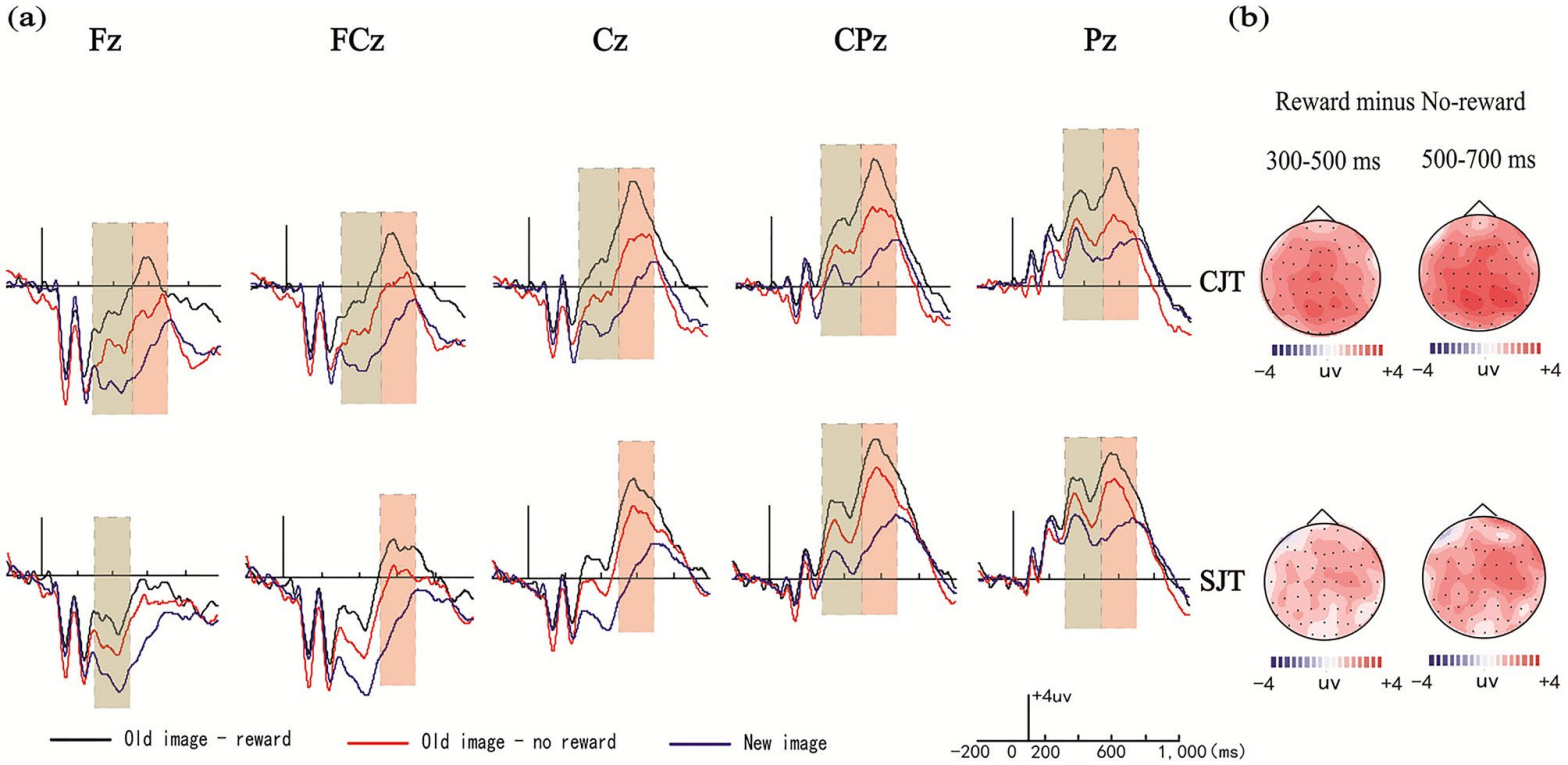
Amplitude distribution and topographic maps of ERPs in the different conditions in the object test phase. (**a**) Amplitudes of the old-rewarded images, the old-unrewarded images and the new images were compared in the congruity-judgment and size-judgment tasks. (**b**) Topographic maps of reward-effect ERPs in the two encoding conditions. Note, CJT, congruity-judgment task; SJT, size-judgment task.

Average amplitudes for each condition during each time window were analyzed by 2 (encoding task: congruity-judgment task vs. size-judgment task) × 3 (image type: old images with reward, old images without reward and new images) × 5 (electrode location: Fz, FCz, Cz, CPz, and Pz) repeated-measures ANOVAs. The amplitude distribution and topographic maps of ERPs in each phase are given in Fig. 4.

##### FN400 (300–500 ms)

ANOVA revealed a significant main effect of image type [*F*_(2, 28)_ = 86.53, *p* < 0.001, *η*_*p*_^2^ = 0.86] and a significant electrode location × image type interaction in the size-judgment task [*F*_(8, 22)_ = 5.21, *p* = 0.013, *η*_*p*_^2^ = 0.54]. Further simple effect analysis showed that significant reward effects of FN400 in the congruity judgment task at all five electrodes (Fz, FCz, Cz, CPz, and Pz) (*ps* < 0.001), and significant reward effects of FN400 in the size judgment task at electrodes Fz, CPz, and Pz (*ps* < 0.01). The results also revealed the significant FN400 old/new effects in the congruity judgment and size judgment tasks at all five electrodes (Fz, FCz, Cz, CPz, and Pz) (*ps* < 0.001).

##### LPC (500–700 ms)

ANOVA revealed a significant main effect of image type [*F*_(2, 28)_ = 43.08, *p* < 0.001, *η*_*p*_^2^ = 0.76], and a significant electrode location × image type interaction in the size-judgment task [*F*_(8, 22)_ = 4.81, *p* = 0.030, *η*_*p*_^2^ = 0.55]. Further simple effect analysis showed that significant reward effects of LPC in the congruity judgment task at all five electrodes (Fz, FCz, Cz, CPz, and Pz) (*ps* < 0.001), and significant reward effects of LPC in the size judgment task at electrodes FCz, Cz, CPz, and Pz (*ps* < 0.01). The results also showed the significant LPC old/new effects in the congruity judgment and size judgment tasks at all five electrodes (Fz, FCz, Cz, CPz, and Pz) (*ps* < 0.001).

Reward difference waves (the average amplitudes in reward conditions minus the average amplitudes in nonreward conditions) were compared, since we found that the reward differences of the congruity-judgment task may be greater than those of the size-judgment task at 300–500 ms and 500–700 ms. The average amplitude differences for each condition during each time window were analyzed by 2 (encoding task: congruity-judgment task vs. size-judgment task) × 5 (electrode location: Fz, FCz, Cz, CPz, and Pz) repeated-measures ANOVAs. The results showed significant main effects of the encoding task at both 300–500 ms and 500–700 ms [*F*_(1, 29)_ = 4.27, *p* = 0.045, *η*_*p*_^2^ = 0.13; *F*_(1, 29)_ = 7.13, *p* = 0.011, *η*_*p*_^2^ = 0.20], which indicated that the amplitude differences of the reward effect (FN400 and LPC) in the congruity-judgment task were greater than those in the size-judgment task in the two windows at all electrodes (Fz, FCz, Cz, CPz, and Pz) (*ps* < 0.05).

#### ERPs in the background (source) test phase

**Figure 5:**
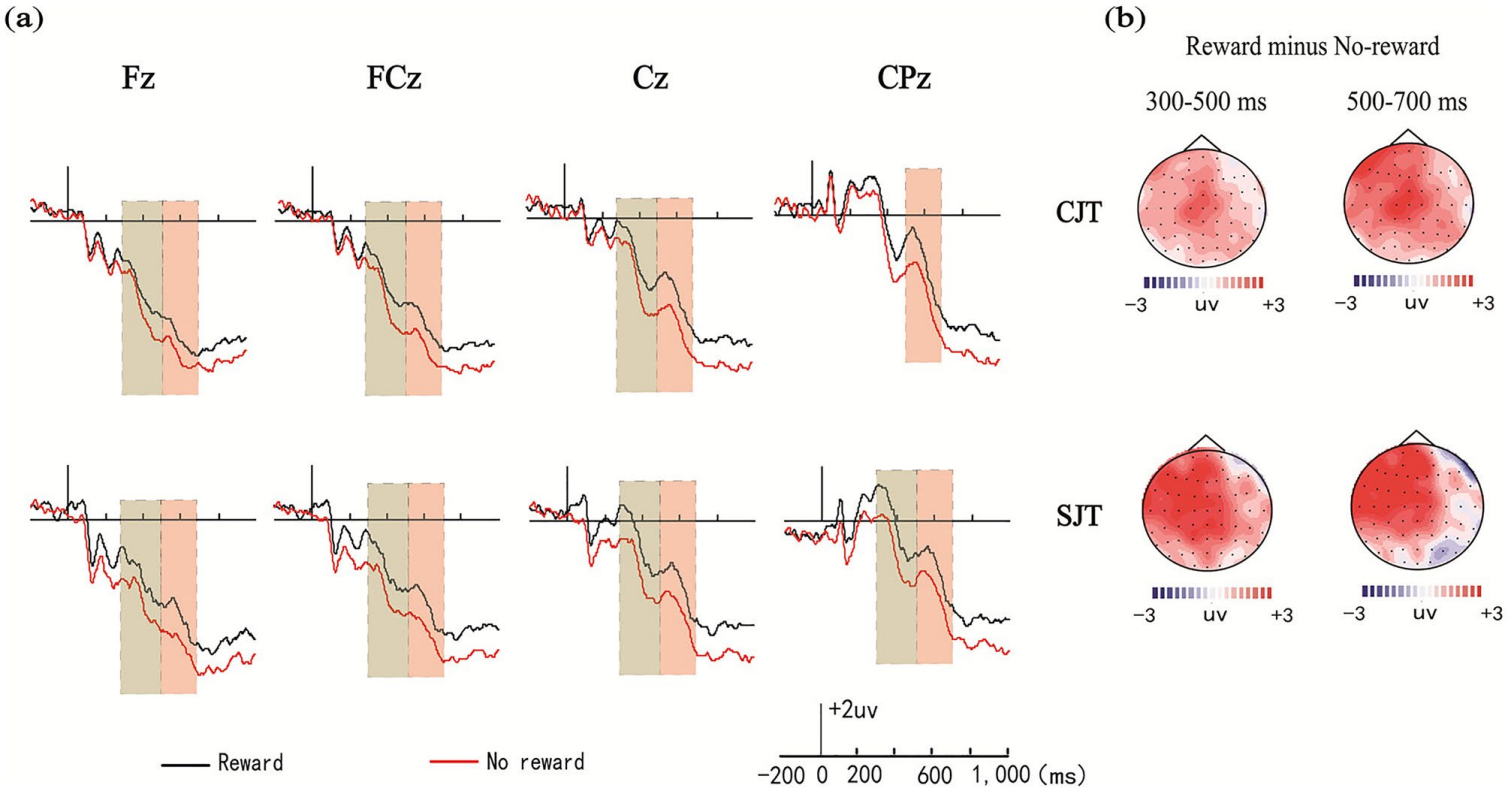
Amplitude distribution and topographic maps of ERPs in the different conditions in the background test phase. (**a**) Amplitudes of the rewarded images and the unrewarded images were compared in the congruity-judgment and size-judgment tasks. (**b**) Topographic maps of reward-effect ERPs in the two encoding conditions. Note, CJT, congruity-judgment task; SJT, size-judgment task.

Average amplitudes for each condition during each time window were analyzed by 2 (encoding task: congruity-judgment task vs. size-judgment task) × 2 (reward type: reward vs. no-reward) × 5 (electrode location: Fz, FCz, Cz, CPz, Pz) repeated-measures analyses of variance (ANOVAs). The amplitude distribution and topographic maps of ERPs in each phase are given in Fig. 5.

##### FN400 (300–500 ms)

ANOVA showed significant main effects of encoding task and reward type [*F*_(1, 26)_ = 8.19, *p* = 0.009, *η*_*p*_^2^ = 0.32; *F*_(1, 26)_ = 26.21, *p* < 0.001, *η*_*p*_^2^ = 0.49] and a significant interaction between reward type and electrode location in the congruity judgment task [*F*_(4, 23)_ = 3.76, *p* = 0.025, *η*_*p*_^2^ = 0.16]. Further simple effect analysis showed significant reward effects of FN400 in the size judgment task at electrodes Fz, FCz, Cz, and CPz (*ps* < 0.001), and significant reward effects of FN400 in the congruity judgment task at electrodes Fz, FCz, and CPz (*ps* < 0.05).

##### LPC (500–700 ms)

ANOVA showed significant main effects of encoding task and reward type [*F*_(1, 26)_ = 5.25, *p* = 0.018, *η*_*p*_^2^ = 0.27; *F*_(1, 26)_ = 13.65, *p* = 0.005, *η*_*p*_^2^ = 0.32] and a significant interaction between reward type and electrode location in the congruity judgment task [*F*_(4, 23)_ = 3.85, *p* = 0.026, *η*_*p*_^2^ = 0.17]. Further simple effect analysis showed significant reward effects of LPC in the size judgment task at electrodes Fz, FCz, Cz, and CPz (*ps* < 0.001) and in the congruity judgment task at electrodes FCz, Cz, CPz, and Pz (*ps* < 0.01).

Reward difference waves were compared since we found that the reward differences of the size-judgment task may be greater than those of the congruity-judgment task at 300–500 ms and 500–700 ms. The average amplitude differences for each condition during each time window were analyzed by 2 (encoding task: congruity-judgment task vs. size-judgment task) × 5 (electrode location: Fz, FCz, Cz, CPz, and Pz) repeated-measures ANOVAs. The results showed a significant main effect of the encoding task [*F*_(1, 29)_ = 3.23, *p* = 0.031, *η*_*p*_^2^ = 0.26] only at 300–500 ms, which indicated that the amplitude differences of the reward effect (FN400) in the size-judgment task were greater than those in the congruity-judgment task at electrodes Fz, FCz, Cz, and CPz (*p* = 0.031).

## Discussion

The current study explored the effects and neural mechanisms of encoding reward anticipation and encoding tasks in item and source memory using event-related potentials (ERPs). This study found that the reward effect of the congruity-judgment task was greater than that of the size-judgment task in item memory and that the reward effect of the size-judgment task was greater than that of the congruity-judgment task in source memory.

In the encoding phase, the ERP results showed that the average amplitudes of the congruity-judgment task were more positive than those of the size-judgment task at 500 ms (LPP). Marini et al^3^. reported that later LPP might reflect memory encoding, and the average amplitudes of items with deeper processing strategies were found to be more positive than those with shallower processing strategies^25^. Accordingly, we suggest that the results indicate that the connections between items and backgrounds in the congruity-judgment task were deeper in the study phase; therefore, the congruity-judgment task trequired more cognitive resources and led to improved source memory performance^3^.

In terms of item memory, the behavioral results showed that the accuracy of rewarded items was significantly higher than that of unrewarded items in the congruity-judgment task and there was also no significant difference in accuracy in the size-judgment task. The response time of the congruity-judgment task was also longer than that of the size-judgment task. Shigemune et al. showed that a reward-related enhancement of memory is found only when memory retrieval is difficult (corresponding to shallow encoding)^13^. Our behavioral results might indicate that item retrieval in the congruity-judgment task may be more difficult than that in the size-judgment task, likely due to increased task complexity. The congruity-judgment task required the participants to judge whether the object matched the background or not, while the size-judgment task asked the participants to judge the size of objects compared with that of the computer screen. We believe that the congruity-judgment task was more complex than the size-judgment task, thus reducing attentional resources to the objects and increasing the time to retrieve items in the congruity-judgment task. The ERP results further showed that the reward effects of FN400 (300–500 ms) and LPC (500–700 ms) in the congruity-judgment task were significantly greater than those in the size-judgment task from electrodes Fz to Pz. These findings support the idea that reward effects for difficult-to-retrieve items are larger than thode for easy-to-retrieve items, which is consistent with both previous studies^13–16^ and the optimal level theory. In addition, we found the FN400 old/new effect (associated with familiarity) and the LPC old/new effect (associated with recollection) in item retrieval, which indicated that the participants possessed both familiarity and recollection during object recognition.

In terms of source memory, the behavioral results showed that the reward difference in the size-judgment task was significantly greater than that in the congruity-judgment task. Additionally, the response time of the size-judgment task was longer than that of the congruity-judgment task, which suggested that the source retrieval of the size-judgment task was more complex and difficult. The ERP results of the encoding phase also confirmed this viewpoint, as the average amplitudes of the congruity-judgment task were more positive than those of the size-judgment task; therefore, the connections between items and source in the congruity-judgment task were deeper, and source retrieval was easier. The ERP results further showed that the reward effects of FN400 (300–500 ms) and LPC (500–700 ms) in the size-judgment task were significantly greater than those in the congruity-judgment task from electrodes Fz to CPz. These source memory results revealed that the reward effects in the size-judgment task (at optimal difficulty) were larger than those in the congruity-judgment task.

Reward significantly improved item recognition accuracy in the congruity-judgment task, and reward played a greater role in the size-judgment task for source recognition accuracy. These results indicated that the reward effect was stronger when the difficulty of retrieval was more complex both in item memory and source memory, which was consistent with our expectations and the results of previous studies^14–16^. Therefore, we believe that both item retrieval and source retrieval focus on rewarded items and occupy more cognitive resources when receiving rewards.

## Conclusion

In the present ERP study, we investigated the effects and neural mechanisms of reward anticipation and encoding tasks during encoding on memory. These item memory results found that the reward effects of FN400 and LPC in the congruity-judgment task were significantly greater than those in the size-judgment task; source memory results found that the reward effect of FN400 and LPC in the size-judgment task were significantly greater than those in the congruity-judgment task. That is, encoding tasks moderated the reward effect at FN400 and LPC, and there was a greater reward effect on recognition tasks that were optimal to retrieve than too difficult or easy to retrieve both in item and source memory.

## Data Availability

The data from the current study are available from the corresponding author upon reasonable request.
